# Clinical predictors of persistence in IgE-mediated cow’s milk allergy in children with multiple food allergies

**DOI:** 10.1007/s11845-026-04417-9

**Published:** 2026-05-07

**Authors:** Ercan Yılmaz, Erdem Topal

**Affiliations:** 1 Faculty of Medicine, Pediatric Allergy and Immunology Department, Inonu University, Malatya, Turkey; 2 Faculty of Medicine, Department of Pediatric Allergy and Asthma, Inonu University, Malatya, 44280 Turkey

**Keywords:** Multiple food allergy, Cow’s milk allergy, Tolerance

## Abstract

**Objective:**

This study aimed to identify independent clinical predictors of failure to develop tolerance to IgE-mediated cow’s milk allergy in children with multiple food allergies.

**Method:**

This retrospective cohort study included 134 children diagnosed with IgE-mediated cow’s milk allergy with multiple food allergies. Patients were divided into two groups based on tolerance development: those who developed tolerance and those who did not. Demographic characteristics, clinical findings after food intake, and laboratory parameters were compared between groups. To identify independent clinical risk factors associated with the lack of tolerance development, clinical variables found to be statistically significant (*p* < 0.05) in univariate analyses were included in multivariate logistic regression analysis.

**Results:**

Ninety-six patients (71.6%) were male, with a median age of 84 months (range: 33–146 months). The median age at symptom onset was 5 months (range: 1–36 months), and the median follow-up duration was 79 months (range: 1–93 months). Atopic eczema was present in 42.5% of patients, and a history of recurrent wheezing or asthma was present in 14.2%. Among concomitant food allergies, egg allergy was the most common (50.7%). While cutaneous findings were most frequently observed in initial reactions, wheezing (18.7%) was the most common respiratory symptom. Anaphylaxis was diagnosed in 24.6% of patients. In the multivariate logistic regression analysis performed to determine the clinical risk factors associated with the failure to develop tolerance to IgE-mediated cow’s milk allergy in patients, the following factors were identified: as the initial reaction after cow’s milk intake are anaphylaxis [OR: 4.95 (1.82–13.5), *p* = 0.002], hypotension [OR: 3.97 (1.19–13.2), *p* = 0.025)], wheezing [OR: 2.86 (1.11–7.36), *p* = 0.029] and lethargy [OR: 3.53 (1.23–10.1), *p* = 0.019)] were identified as independent risk factors.

**Conclusion:**

Severe clinical reactions initially presenting with systemic and respiratory involvement are independent clinical predictors of failure to develop tolerance to IgE-mediated cow’s milk allergy in children with multiple food allergies.

## Introduction

Cow’s milk allergy is one of the most common food allergies in childhood and usually appears in the first months of life [1, 2]. Although a significant proportion of children with IgE-mediated cow’s milk allergy develop tolerance over time, the disease can be more severe and tolerance development may be delayed, especially in cases accompanied by multiple food allergies [3, 4]. This patient group carries a higher risk of persistent allergy, making early identification of the risk phenotype crucial for effective clinical management.

The literature has shown that laboratory parameters such as serum food-specific IgE levels and skin prick tests are valuable in predicting the development of tolerance [5–7]. However, due to the heterogeneity of the immune response in children with multiple food allergies, the prognostic power of these parameters may be limited, and these tests may not be readily available in every clinical setting [3, 6]. This situation highlights the importance of clinically based predictors, independent of laboratory data, particularly in IgE-mediated cow’s milk allergy accompanied by multiple food allergies.

In IgE-mediated food allergy, the severity and systemic spread of the allergic reaction that develops after the first exposure provide important clues about the natural course of the disease [8, 9]. Severe clinical findings such as anaphylaxis and cardiovascular and respiratory system involvement may reflect a more persistent and resistant allergic phenotype. However, in IgE-mediated cow’s milk allergy accompanied by multiple food allergies, the role of initial clinical reaction findings in predicting tolerance development is not sufficiently clear.

In this study, we aimed to evaluate the relationship between clinical reaction findings observed during the first exposure to cow’s milk in children with multiple food allergies and diagnosed with IgE-mediated cow’s milk allergy, and the results of oral food provocation tests and the development of tolerance. The goal was to create a model based solely on clinical findings, excluding laboratory tests; thus, the aim was to identify predictors in the early stages that are easy to apply and can directly contribute to clinical practice.

## Materyal ve method

### Study design and data collection

This retrospective observational study was conducted at the Pediatric Allergy and Immunology Clinic of a tertiary referral center. Children who underwent oral food challenge (OFC) between January 2017- January 2024 year for immunoglobulin E (IgE)–mediated cow’s milk allergy with accompany other food allergy were included in the study. Children allergic to two or more foods were defined as having multiple food allergies. Patients were eligible if they had a clinical history suggestive of an immediate-type allergic reaction to cow’s milk and other food and evidence of IgE sensitization, defined as a positive skin prick test (SPT) and/or elevated food-specific serum IgE levels. Patients with non-IgE–mediated food allergy, mixed-type reactions, food protein-induced enterocolitis syndrome (FPIES), eosinophilic gastrointestinal disorders, or incomplete clinical data were excluded.

Patients’ age, gender, age at diagnosis, follow-up periods, breastfeeding, comorbid atopic diseases, exposure to cigarette smoke, pet ownership, family history of atopic disease, names of multiple foods to which they developed allergies, reactions initially developed to cow’s milk, laboratory tests, and oral food challenge (OFC) test results were recorded in their files. Patients were divided into two groups according to OFC outcome: OFC-positive and OFC-negative. Demographic characteristics, laboratory findings, and allergic reactions initially developing to cow’s milk were statistically compared between the two groups. Parameters that were statistically significant were included in the logistic regression model to determine whether risk factors for the failure to develop tolerance were associated with the allergic reactions initially developing.

### OFC protocol

At our clinic, OFC tests are performed under doctor supervision, after all anaphylaxis precautions have been taken and written consent has been obtained from the patient and/or parent. Patients are examined before starting OFC and before each dose increase. Vital signs, respiratory and skin examination findings are recorded in the patients’ files after each dose. Any reactions that develop after the doses are administered are also recorded in their files. Patients are kept under observation for two hours after the last dose is administered. In OFC tests at our clinic, the suspected food is started at a low dose, and dose increases are made at 15-minute intervals until the target dose is reached. In our clinic, the starting doses and dose increments for OFCs for each food are applied in accordance with the American Academy of Allergy, Asthma, and Immunology’s “Work Group Report: Oral Food Challenge Testing” guidelines [10]. If objective or recurrent subjective findings are present during the OFC, the test is considered positive [10, 11]. Parents were instructed to notify the researcher if any delayed reactions occurred after discharge.

Medications that could affect test results, such as antihistamines or corticosteroids, were discontinued prior to OFC. In addition, accompanying conditions such as asthma, uncontrolled urticaria or eczema, or concurrent respiratory tract infection were evaluated before OFC was performed.

### Laboratory evaluation

Serum total IgE level, eosinophil count and specific IgE level for related food were measured. Specific IgE level > 0.35 kUA /L for any allergen was considered positive. Those with an absolute eosinophil count greater than 500/mm³ in complete blood count was considered eosinophilia [1, 8, 12].

### Skin prick test

Skin prick test (SPT) was performed with the standard commercial extracts or via skin prick to prick technique for non-available extracts on the volar surface of the forearms or on the back of the body with a 1-mm lancet according to EAACI guidelines [13]. A positive SPT was defined as a mean wheal diameter 3 mm larger than the negative control. Histamine (10 mg/mL) and saline were used as positive and negative controls, respectively. The commercial food allergen extracts (Abello-ALK, Madrid, Spain) used for the SPT were cow’s milk, hen’s egg (white and yolk), beef, chicken, fish mix, wheat, rice, soybean, lentil, hazelnut, walnut, peanut, and pistachio.

### Statistical analysis

Statistical analysis was performed with SPSS Statistics (v.21.0; IBM Corp., Armonk, NY) and Microsoft Excel (Microsoft Corp., Redmond, WA). Normality was evaluated by the Kolmogorov-Smirnov and Shapiro-Wilk tests. Descriptive statistics were expressed as the frequency and percentage for categorical variables, whereas the median (min-max) was used for nonnormally distributed quantitative data. The categorical variables were compared by using the Pearson x^2^ test or the Fisher exact test, and the quantitative variables were compared with the Mann–Whitney U test. The logistic regression model was constructed to include only clinical reaction findings that developed during the patient’s first exposure to cow’s milk. Statistically significant clinical findings were included in the multivariate logistic regression analysis. A two-sided p value of < 0.05 was considered statistically significant.

The research protocol was approved by the ethical committee of Non-Invasive Clinical Research of Inonu University, Turkey (Approval number: 2025/6111). The families of the participants provided written informed consent.

## Results

The study included a total of 134 children diagnosed with IgE-mediated cow’s milk protein allergy and concomitant multiple food allergies. Ninety-six (71.6%) of the patients were male, and the median age was 84 months (range 33–146 months). The median age of symptom onset was 5 months (range 1–36 months), and the median follow-up duration was 79 months (range 1–93 months). During follow-up, 105 patients (78.4%) developed tolerance to cow’s milk, while 29 patients (21.6%) did not. Atopic eczema was present in 57 (42.5%) patients, and recurrent wheezing/asthma in 19 (14.2%) patients. Among the concomitant food allergies, egg allergy was the most common [68 patients (50.7%)], followed by peanut [19 patients (14.1%)], meat [4 patients (2.9%)], lentil [3 patients (2.2%)] and walnut [1 patient (0.7%)] allergies. Laboratory evaluation revealed a median milk-specific IgE level of 5.6 kUA/L (0.1–268), a median eosinophil count of 540/mm^3^ (10–3800/mm^3^), and a median highest induration diameter in skin prick test was 3 mm (0–15). The demographic characteristics and laboratory findings of the patients are summarized in Table 1. When comparing the group that developed tolerance with the group that did not, there was no statistically significant difference in terms of the demographic characteristics of the patients (*p* > 0.05). In terms of laboratory findings, the milk-specific IgE level (median value: 12.9 kUA/L vs. 5.2 kUA/L; *p* = 0.002) and the diameter of the reaction in the skin prick test (median induration diameter: 4 mm vs. 3 mm; *p* = 0.0016) were significantly higher in the group that did not develop tolerance (Table 1).

**Table 1:** Demographic and Laboratory Findings in IgE-Mediated Cow’s Milk Allergy in Children with Multiple

Demographics	All patients (*n*: 134) *n* (%)	OFC negative group (*n*: 105) *n* (%)	OFC positivity group (*n*:29) *n* (%)	*p* value
Gender, male	96 (71.6)	74 (70.5)	22 (75.9)	0.57
Age, median (min-max), month	84 (33–146)	84 (33–146)	84 (48–120)	0.88
Age of onset of symptoms, median (min-max), month	5 (1–36)	5 (1-136)	4 (2–13)	0.64
Follow up duration, median (min-max), month	79 (1–93)	80 (1–37)	79 (42–114)	0.91
Other co-atopic diseases				
Atopic eczema	57 (42.5)	49 (46.7)	8 (27.6)	0.08
Recurrent wheeze/Asthma	19 (14.2)	15 (14.3)	4 (13.8)	0.94
Allergic rhinitis doctor diagnosed	3 (2.2)	2 (1.9)	1 (3.4)	0.42
Family atopy history				
Asthma	78 (58.2)	57 (54.3)	21 (72.4)	0.11
Allergic rhinitis	83 (61.9)	61(58.1)	22 (75.9)	0.11
Food allergy history	15 (11.2)	13 (12.4)	2 (6.9)	0.53
Drug allergy	2 (1.5)	1 (1)	1 (3.4)	0.42
Other food allergies				
Egg	68 (50.7)	51 (48.5)	17 (58.6)	0.33
Peanut	19 (14.1)	15 (14.2)	4 (13.9)	1
Lentil	3 (2.2)	2 (1.9)	1 (3.4)	0.52
Meat	4 (2.9)	2 (1.9)	2 (6.8)	0.2
Walnut	1 (0.7)	1 (0.9)	-	1
Breast feeding ≥ 6 months	95 (70.9)	78 (74.3)	17 (58.6)	0.29
Pet in home	7 (5.2)	4 (3.8)	3 (10.3)	0.21
Exposure to cigarettes	16 (11.9)	14 (13.3)	2 (6.9)	0.37
Laboratory				
Cow’s milk specific Ig E, median (min-max), kUA/L	5.6 (0.1–268)	5.2 (0.1–268)	12.9 (0.1–100)	0.002
Eosinophilia, median (min-max), cell/mm^3^	540 (10-3800)	560 (10-2190)	490 (20-3800)	0.57
The highest induration diameter in skin prick test, median (min-max), mm	3 (0–15)	3 (0–15)	4 (0–10)	0.016

The most common initial reactions in IgE-mediated cow’s milk allergy were cutaneous symptoms (itching in 72.4% of patients, flushing around the mouth in 61.9%, angioedema in 53%, and urticaria in 44.8%). Among gastrointestinal symptoms, vomiting was the most common [17 patients (12.7%)], and among respiratory symptoms, wheezing was the most common [25 patients (18.7%)]. Anaphylaxis was diagnosed in 33 (24.6%) patients. In the group that did not develop tolerance, the rates of skin, respiratory, and gastrointestinal involvement and anaphylaxis diagnosis were higher than those in the group that developed tolerance. The initial symptoms of the patients and the comparison of the two groups are summarized in Table 2.

**Table 2:** Initial symptoms of IgE mediated cow’s milk allergy in children with multiple food allergies

Demographics	All patients (*n*: 134) *n* (%)	OFC negative group (*n*: 105) *n* (%)	OFC positivity group (*n*:29) *n* (%)	*p* value
Cutaneous				
Pruritus without skin lesions	97 (72.4)	71 (67.6)	26 (89.7)	0.09
Flashing around the mouth	83 (61.9)	59 (56.2)	24 (82.8)	0.018
Urticaria	60 (44.8)	37 (35.2)	23 (79.3)	< 0.001
Angio-edema	71 (53)	47 (44.8)	24 (82.8)	< 0.001
Gastrointestinal				
Vomiting	17 (12.7)	9 (8.6)	8 (27.6)	0.011
Dysphagia	7 (5.2)	2 (1.9)	5 (17.2)	0.005
Gastro-esophageal reflux	6 (4.5)	2 (1.9)	4 (13.8)	0.02
Respiratory				
Cough	24 (17.9)	14 (13.3)	10 (34.5)	0.012
Dyspnea	18 (13.4)	11 (10.5)	7 (24.1)	0.048
Stridor	13 (9.7)	7 (6.7)	6 (20.7)	0.029
Wheezing	25 (18.7)	14 (13.3)	11 (37.9)	0.003
Rhinitis symptoms	15 (11.2)	8 (7.6)	7 (24.1)	0.021
Cardiac				
Hypotension	7 (5.2)	2 (1.9)	5 (17.2)	0.002
Tachycardia	7 (5.2)	2 (1.9)	5 (17.2)	0.002
Neurological				
Fainting	3 (2.2)	1 (1)	2 (6.9)	0.12
Lethargy	9 (6.7)	3 (2.9)	6 (20.7)	0.001
Anaphylaxis	33 (24.6)	17 (16.2)	16 (55.2)	< 0.001

In a multivariate logistic regression analysis conducted to determine clinical risk factors associated with the failure to develop tolerance to IgE mediated cow’s milk allergy; the initial reaction being anaphylaxis [OR: 4.95 (1.82–13.5), *p* = 0.002], hypotension [OR: 3.97 (1.19–13.2), *p* = 0.025)], wheezing [OR: 2.86 (1.11–7.36), *p* = 0.029] and lethargy [OR: 3.53 (1.23–10.1), *p* = 0.019)] after cow’s milk intake were identified as independent risk factors (Table 3). In contrast, cutaneous findings such as urticaria and angioedema, as well as cough lost their significance in the multivariable logistic regression model and were not independently associated with the lack of tolerance development.

**Table 3:** Multivariable logistic regression analysis of predictors for IgE mediated cow s milk allergy for intolerance

Predictor*	β (Beta)	OR (95%CI)	*p* value
Anaphylaxis	1.60	4.95 (1.82–13.5)	0.002
Hypotension	1.38	3.97 (1.19–13.2)	0.025
Wheezing	1.05	2.86 (1.11–7.36)	0.029
Lethargy	1.26	3.53 (1.23–10.1)	0.019

*Anaphylaxis, hypotension, wheezing, cough, urticaria, angioedema, and lethargy were simultaneously entered into the multivariable logistic regression model to identify independent predictors of tolerance persistence

## Discussion

In this study, it was demonstrated that the clinical characteristics of the initial allergic reaction independently predicted the failure to develop tolerance for IgE-mediated cow’s milk allergy in children with multiple food allergies. In the multivariate logistic regression analysis, anaphylaxis, hypotension, wheezing, and lethargy were identified as the strongest independent risk factors for the failure to develop tolerance, swa.

Our study was specifically designed to exclude laboratory findings from the logistic regression analysis, as the predictive value of specific IgE levels and other laboratory parameters for oral food challenge test results is well established in the literature [5–7, 14]. The primary objective of the study is to evaluate whether the positivity of the oral food challenge test and, consequently, the development of tolerance can be predicted based solely on the clinical reaction findings at the patient’s initial presentation. In this regard, a logistic regression model was constructed to include only the initial clinical symptoms, and it was investigated whether risk classification was possible using objective, easily accessible clinical data. The findings indicate that the severity and systemic spread of the initial reaction can provide meaningful prognostic information regarding oral food challenge test results and tolerance development without the need for laboratory tests. This approach may contribute to determining patient selection and follow-up strategies, particularly in clinical settings where resources are limited or laboratory tests are not always accessible.

Among all variables, anaphylaxis emerged as the strongest predictor, increasing the risk of failure to develop tolerance by approximately fivefold. This finding suggests that an initial severe allergic reaction reflects a stronger and more persistent IgE-mediated immune response, which may negatively affect tolerance development. The literature also reports that tolerance development is delayed in cases presenting with anaphylaxis or multisystem involvement [5, 6, 15].

Hypotension, an indicator of cardiovascular involvement, has also been found to negatively affect tolerance development as an independent risk factor. This suggests that objective findings reflecting systemic circulatory dysfunction may provide additional prognostic information beyond the definition of anaphylaxis [8, 9]. Similarly, wheezing, which indicates lower airway involvement, has been identified as a factor negatively affecting tolerance development, highlighting the importance of respiratory system involvement in disease severity and tolerance development in long-term follow-up.

Lethargy has been identified as another significant independent predictor of failure to develop tolerance. Lethargy, which is a sign of systemic involvement and possible hypoperfusion or hypoxia associated with hypotension during the acute reaction, may reflect a higher inflammatory load and widespread mediator release [8, 9].

In contrast, cutaneous findings such as urticaria and angioedema, as well as cough, were not independently associated with the lack of tolerance development in the multivariable logistic regression model. These findings suggest that isolated or mild organ involvement may not adequately reflect the underlying immunological processes determining tolerance development.

Overall, our results indicate that tolerance development in IgE-mediated cow’s milk allergy in children with multiple food allergies is associated with the severity and systemic spread of the initial clinical picture rather than the presence of individual symptoms.

Clinically, early identification of children presenting with anaphylaxis, hypotension, wheezing, or lethargy may contribute to the identification of patients at high risk for persistence. This approach may assist in individualizing decisions regarding the planning of follow-up frequency, family education, and the timing and safety of oral food challenge tests.

This study has some limitations. The retrospective design may lead to information bias in the recording of clinical findings. However, the fact that all cases were evaluated at a tertiary pediatric allergy center using standard diagnostic criteria has reduced the possibility of misclassification. Furthermore, the limited sample size may restrict the generalizability of the findings. Nevertheless, the consistency and statistical power of the obtained relationships suggest that the results are clinically meaningful.

The failure to develop tolerance to IgE-mediated cow’s milk allergy in children with multiple food allergies is largely related to the severity and systemic nature of the initial allergic reaction. The presence of anaphylaxis, cardiovascular involvement, wheezing, and lethargy in the presentation identifies a high-risk patient group that may require closer monitoring and individualized management.
